# Detection and characterization of purified antigenic proteins from culture filtrate of *Mycobacterium bovis* strain AN5

**Published:** 2020-02

**Authors:** Malihe Honarmand Kashi, Nader Mosavari, Mitra Salehi, Naheed Mojgani

**Affiliations:** 1 Department of Biology, Islamic Azad University of North Tehran Branch, Tehran, Iran; 2 Department of PPD Tuberculin, Razi Vaccine and Serum Research Institute, Agriculture Research, Education and Extension Organization, Karaj, Iran

**Keywords:** *Mycobacterium bovis*, Purified antigenic proteins, Dot-blot, Western blot

## Abstract

**Background and Objectives:**

Bovine tuberculosis diagnosis is usually performed by various tests with specific limitations. *Mycobacterium bovis* culture filtrate contains antigenic proteins that could be used to improve the sensitivity of bovine tuberculosis diagnosis. The objective of this study was to identify and purify antigenic proteins from culture filtrates of *M. bovis* strain AN5 for use in immunological assays.

**Materials and Methods:**

Secreted proteins were purified from the heat-treated culture filtrate of *M. bovis* strain AN5. Proteins were precipitated with ammonium sulfate, fractionated by Sephadex G50 chromatography. The protein concentrations and the approximate molecular weight were determined by lowry method and 12% sodium dodecyl sulfate polyacrylamide gel electrophoresis (SDS-PAGE), respectively. Immunological methods, including dot-blotting and western blotting, assessed the quality of the isolated proteins.

**Results:**

The quantity of antigenic proteins in the culture medium was measured at far more than 15% of the amount of proteins secreted into medium. Three main chromatographic fractions obtained and showed concentrations of proteins ranging from 14 to 60 μg/ μl with molecular weights in the 10 to 180 kDa range. The purified antigens showed positive reactions to the infected cattle serum throughout dot-blotting. Western blotting revealed a total of 15 to 70 kDa molecular weight proteins.

**Conclusion:**

Immunoblotting analysis made it possible to detect and recognize novel antigens that are useful for bovine tuberculosis diagnosis improvement. This is significant since non-specific reactions were not observed when we utilized serum of cattle experimentally infected with *M. bovis* as a polyclonal antibody.

## INTRODUCTION

*Mycobacterium bovis* is the main causative agent of bovine tuberculosis that causes zoonotic disease and infection in a wide range of hosts including farmed animals, wildlife and humans (1, 2). This infection causes trade barriers and financial problems to farming economies worldwide. The disease control programs held in major countries include detection of infected animals by the tuberculin skin test (TST) and elimination of the infected animal (3).

Purified protein derivative (PPD), which is purified from the heated culture filtrates of mycobacteria, is a specific antigen mainly and widely used for tuberculosis diagnosis and immunological investigations (4). However, because humoral antibody immunity tests are less sensitive to bovine tuberculosis than cell-mediated immunity test, development of more specific reagents for the diagnosis of *M. bovis* infection is essential (5, 6 and 7). To date, a number of mycobacterial proteins have been investigated, isolated, cloned, purified and distinguished by different techniques. Some of these antigens include mycobacterial protein bovis 83 (MPB83), mycobacterial protein bovis 64 (MPB64), early secretory antigenic target 6 (kDa) (ESAT-6) and culture filtrate protein (CFP-10). These antigens are known to cause T helper 1 (Th1) cell response that releases proinflammatory cytokines including interferon-γ (IFN-γ) (8–12).

The low molecular weight proteins mainly ESAT-6 and CFP-10 are significantly immunogenic and have the potential to enhance the sensitivity of diagnosis without reducing specificity in *M. bovis* BCG-vaccinated communities. Moreover the genes for both mentioned proteins have been detected in *M. tuberculosis, M. africanum* and virulent *M. bovis*, despite that they are not present in *M. bovis* BCG and in many environmental as well as non-tuberculous mycobacteria (12–14).

A number of techniques have been used for purification of these proteins. Several reports indicated the use of ion-exchange chromatography for separation of these proteins from mycobacterial filtrates. In 1970, Bennedsen applied unheated culture filtrate and a saline cell extract of *M. tuberculosis* to chromatography on diethyl amino ethyl (DEAE)-Sephadex columns, after precipitation with 80% saturated ammonium sulfate (15). Glenchur and his co-workers (16) used Sephadex G-25 chromatography for isolation of antigenic protein. Similarly, in Daniel and Ferguson study (17) two proteins were obtained from *M. tuberculosis* culture filtrates by combined ammonium sulfate precipitation, gel filtration with high-porosity P-300 acrylamide gel.

This work aimed at isolating and purifying antigenic proteins from culture filtrates of *M. bovis* AN5 and using immunological approaches including dot-blotting and western blotting.

## MATERIALS AND METHODS

**Bacterial cultures and antigen preparations.** *M. bovis* strain AN5, ATCC35726 (provided by Vaccine and Serum Research Institute, Karaj, Iran) was used in this study. The bacteria were initially grown on Löwenstein-Jensen medium at 37 °C for 56 days. Bacteria grown on Löwenstein-Jensen medium were incubated in synthetic Dorset-Henley broth medium at 37 °C for five weeks, *M. bovis* AN5 were then transferred and incubated for further cultivation in liquid Dorset-Henley at 37 °C and harvested at 8 weeks, without shaking (18).

**Isolation and purification of antigens from culture fluids.** To isolate the target proteins, *M. bovis* AN5 cultures were heat-treated for 1 h at 68 °C and bacterial particles separated by Buchner funnel and filtrated by 0.45 and 0.22 um (Millipore, USA) filters. Through adding solid ammonium sulfate (at 40% concentration), the proteins in the cell-free culture filtrates were precipitated. Upon centrifugation at 10,000 × g for 10 min, the precipitates were collected and suspended in phosphate-buffered saline (PBS). The salt was removed by dialysis for 48 h at 4 °C in Tris buffer (pH8.7) (3).

**Separation of protein fractions by Sephadex G-50 chromatography.** The desalted protein fractions were added to diethylaminoethyl Sepahadex G50 column (2 cm diameter and 150 cm height) equilibrated with 30 mM Tris buffer, pH 8.7. The gel was washed with 500 ml of phosphate-buffered saline (PBS) (19). The protein concentration of the collected fractions was estimated spectrophotometrically at 280 nm. Fractions were pooled by their concentrations of proteins based on obtained OD, and finally dialyzed against PBS. All fractions were kept at −4 °C for further use.

**Protein concentrations and molecular weight.** The Lowry method evaluated protein concentrations (20) and 12% Sodium dodecyl sulfate polyacrylamide gel electrophoresis (SDS-PAGE) determined the molecular weight of the fractions. For SDS-PAGE, the antigens were diluted in loading buffer and were heated in boiling water for 5 min (6). Twenty μl of protein samples and 4 μl of prestained protein ladder (Sinaclon) in the range of 10 to 250 kDa were loaded to each well. The SDS-PAGE conducted at 80V for 2 h. The separated proteins were stained with Coomassie brilliant blue and destained with 0.04% acetic acid.

**Preparation of positive sera from infected cattle.** The cattle were infected with *M. bovis* AN5 strain and their sera collected for immunological assay. Briefly, approximately 200 mg bacillary powders were mixed with 400 mg of pumice added and moistened with two drops of water and thoroughly grounded with the pestle. A few drops of sterile paraffin were added and the grinding procedure repeated. The obtained suspension (4 mg/ml of bacteria) was injected intramuscularly (1 ml) per cattle. Another injection was done after 1 week and later 2 weeks of second injection. The positive sera were collected after 2 weeks of last injection.

**Dot-blotting.** For dot blots, the Polyvinylidene difluoride (PVDF) membrane was activated in methanol (10 seconds), distilled water (1 min) and TBS (Tris Buffer saline, pH 8) with 10% Tween 20 for 10 min, respectively. The antigens (10 μl of protein per sample) were spotted on the membrane and were dried at room temperature for 1 h. For fixation, the membranes were placed in methanol (10 seconds), and TBST (1 min), respectively, and later blocked for 1 h in 3% (w/v) bovine serum albumin (BSA) under constant shaking. The polyclonal antisera were utilized as primary antibodies (positive serum sample collected from infected cattle with *M. bovis* AN5), while serum from non-infected cattle were used as negative control. The serum samples were diluted 1/50 with 3% (w/v) BSA. The membrane was washed thrice with 1 × TBST in shaking condition. After washing, the secondary antibody (anti-bovine antibody conjugated to peroxidase (Goat pAb to Cow IgG) diluted 1:1000 with 1% (w/v) BSA) was placed on the spots and incubated for 1 h at room temperature, with constant shaking (65 rpm). After another wash cycle, the membrane was reacted and detected by adding DAB (3, 3′-diaminobenzidine) in 10 mL TBST and 500 μL of H_2_O_2_ (30%) for 5 to 15 min in the dark room (21). The test was performed twice to check reproducibility of the results.

**Western blotting or immunoblotting.** In this method, first electrophoresis was carried out on SDS-PAGE. The PVDF membrane was activated in methanol, and then TBST. The antigens were transferred from the gel onto the membrane electrophoretically at 400 mA for 2 h (22, 23). The membrane was activated in methanol and distilled water respectively. After blocking (1 h) with 3% (w/v) BSA, the membrane was incubated with the primary polyclonal antibodies (infected cattle serum with *M. bovis* AN5 at room temperature for 1 h. Serum from non-infected cattle was used as negative control. The membrane was washed in TBST, and immersed and incubated with the secondary antibody (anti-bovine antibody conjugated to peroxidase (Goat pAb to Cow IgG) diluted 1:1000 with 1% (w/v) BSA) for 1 h at room temperature with gentle agitation. After another wash cycle, the membrane was detected and developed by adding DAB in 10 mL TBST and 500 μL of H_2_O_2_ (30%). DAB was used as a substrate and incubated for 5 to 15 min in the dark room. The enzyme reaction was stopped by soaking the membrane in water (22–24).

## RESULTS

**Fig. 1.**
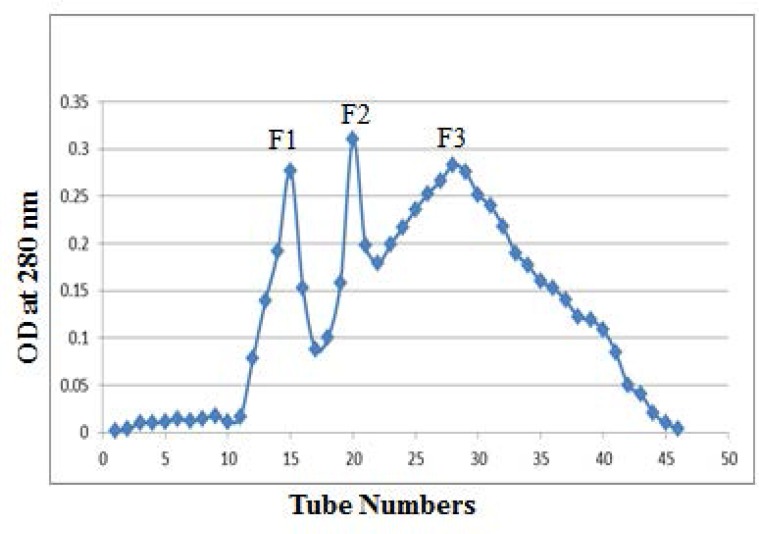
Elution profile through chromatographic separation of the sample on a Sephadex G50 column with a bed volume of 210 ml. The protein concentration in each fraction was determined by the Lowry method. 50 μl sample of each of the eluted fraction was analyzed by SDS-PAGE. Eluted fractions were pooled into three major fractions (F1 to F3).

According to the obtained data, by the addition of ammonium sulfate at 40% saturation, the antigenic proteins were isolated and concentrated from the culture filtrate of *M. bovis* AN5. Based on their molecular size, chromatographic procedure was further purified these proteins. Forty six chromatographic fractions were collected on Sephadex G50 using a whole-gel elutor. The protein concentrations of these fractions were determined using Lowry method. Fig. 1 shows the elution profile after the chromatographic fractionation of *M. bovis* AN5 culture filtrate on a Sephadex G50 column. The eluted fractions were pooled consecutively on the basis of protein concentrations to attain three main fractions named F1, F2 and F3.

**Fig. 2.**
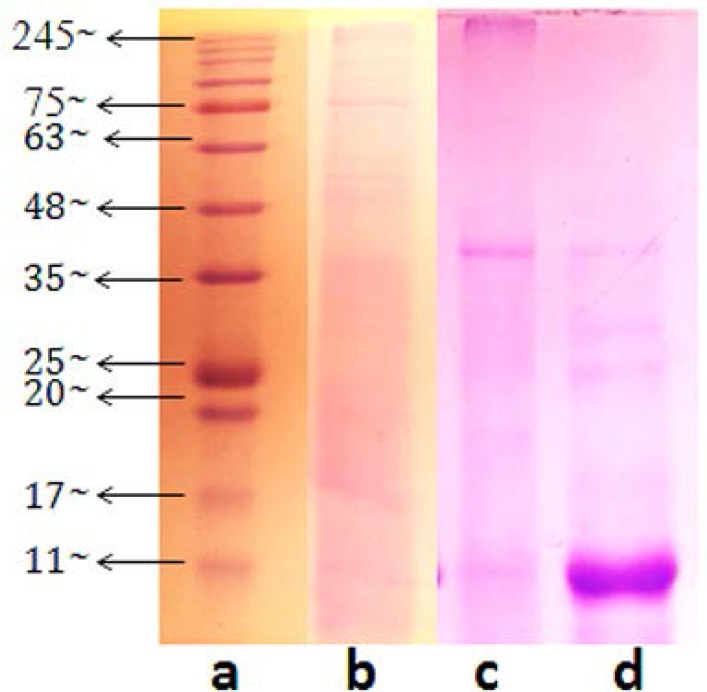
Coomassie brilliant blue stained SDS-PAGE gel of the three fractions; Lane a, prestained protein ladder, (Sinaclon), 10–250 kDa; lane b, the first fraction (F1); lane c, the second fraction (F2); lane d, the third fraction (F3). The F3 fraction was in wide range of 10 to 40 kDa that included a brilliant valuable band in low molecular region.

Also according to the data, approximately F1, F2 and F3 contained proteins at concentrations of 14.386 μg/ μl, 29.186 μg/ μl and 57.32 μg/ μl, respectively. Diffuse and faint bands in the range of 50 to 100 kDa and 30 to 50 kDa in the F1 and F2 fractions respectively observed during molecular size estimations on SDS-PAGE analysis (Fig. 2). Proteins in a wider range of 10 to 40 kDa were included in the F3 fraction. Compared to the other two fractions, the F3 fraction showed the most pronounced and the lowest molecular weight proteins.

**Fig. 3.**
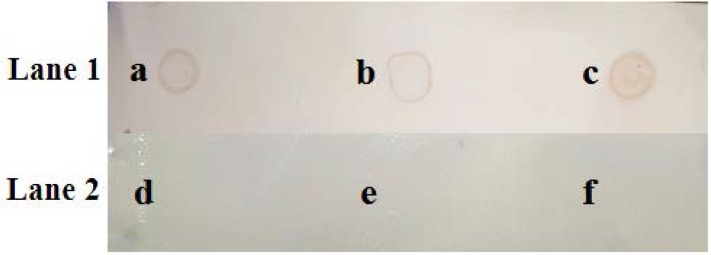
The dot blots of PVDF membrane that 10 μl of protein per sample was spotted on it. The blots were (a and d) undiluted F2 fraction; (b and e) F3 fraction diluted with distilled water (1/2) and (c and f) Undiluted F3 fraction; while Lane 1 were incubated with positive serum as the primary antibody and Lane 2 were the samples reacted with negative serum samples. Anti-bovine antibody conjugated to peroxidase was used as secondary antibody.

Dot-blot was used to distinguish the mycobacterial antigen in the two purified chromatographic fractions (F2 and F3). The F3 fraction was further diluted based on their higher protein concentrations (b and e) and tested with positive and negative serum samples. As seen in Fig. 3, the blots a (F2), b (diluted F3) and c (undiluted F3) showed positive results when treated with positive serum samples. The Dot-blot reliability was assessed using negative serum samples that were obtained from healthy cattle. As expected d, e and f appeared negative.

**Fig. 4.**
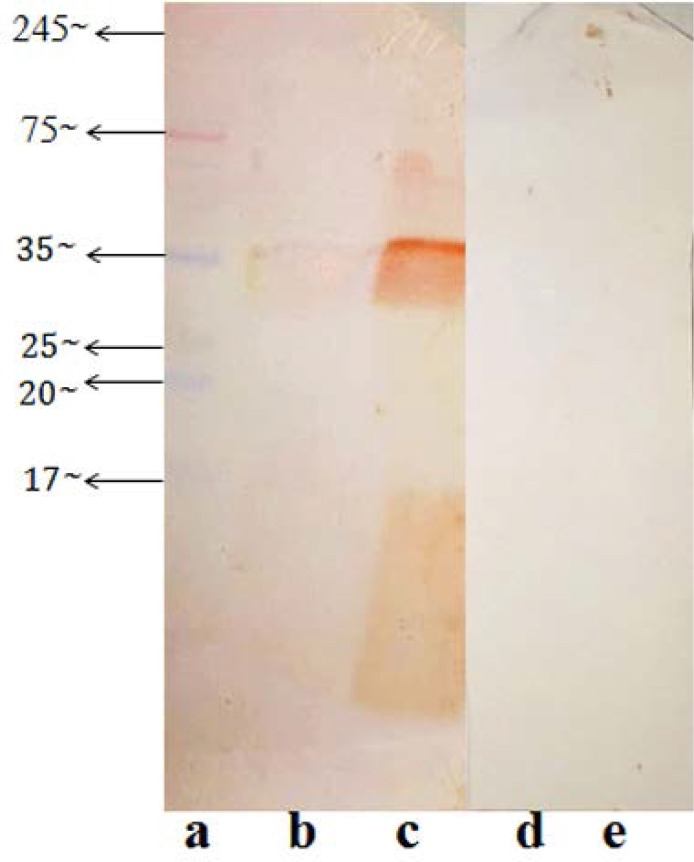
Immunoblot analysis of the second and third fraction (F2 and F3); lane a, SDS-PAGE prestained protein ladder (Sinaclon); lane b F2; lane c, F3; positive serum diluted (1:50 with 1% (w/v) BSA block solution) was utilized as polyclonal primary antibody and anti-bovine antibody conjugated to peroxidase (Goat pAb to Cow IgG) diluted 1:1000 with 1% (w/v) BSA block solution was used as secondary antibody; lane d, F2 with negative serum as primary antibody; lane e, F3 with negative serum as primary antibody.

To characterize the immunogenicity of the purified proteins, western blot assay was performed. Fig. 4 illustrates immunoblotting with polyclonal antibody. According to the results, F3 fraction showed blots in the range of 10 kDa to 40 kDa, while a sharp band was seen at 40 KDa. Similarly, F2 fraction also showed a sharp and dominant band of 40 kDa. No additional bands were seen in this fraction. However, in F3 fraction faint smear in the range below 20 kDa was observed indicating the presence of low molecular weight antigens. No bands were perceived on negative controlled membrane.

## DISCUSSION

Bovine tuberculosis in cattle and wildlife and its operative control are ultimate significance and can be attained through various diagnostic tests. The early stages of the disease were identified by both the tuberculin skin test and the *in vitro* IFN-assay, but do not identify anergic animals and there had been a lack of sensitivity and specificity in both. Histopathology's limitation is that it is carried out postmortem. Furthermore heterogeneity in the existence of lesions between various BTB-infected animal species makes diagnosis difficult. The cultivation of the organism from infected tissues is still considered as the ' gold standard ' procedure for identifying *M. bovis*, followed by clarification using PCR. Because of the slow growth rate of *Mycobacterium*, it takes a long time to produce a result, which is not practical for field testing. Serological tests are utilized to identify the host antibody response to mycobacterial antigens including MPB83, ESAT-6 and CFP-10. These rapid tests have demonstrated great potential for identifying bovine tuberculosis in various species. In the whole blood IFN-γ assay for cattle and humans, low molecular weight proteins including ESAT-6 and CFP-10 have been shown to be magnificent diagnostic target proteins, especially important due to the absence of their genes from *M. bovis* BCG allows for distinction between individuals infected and vaccinated. IFN-γ-based tests utilizing specific mycobacterial antigens have the opportunity of improved specificity particularly in comparison to PPD-based IFN-γ-based tests, but the sensitivity of these tests needs to be enhanced at the moment (11, 12, 22, 24). The focus of present study is to define potential low molecular weight antigens that can be used in serological tests and to improve the sensitivity and specificity of each of these tests.

There are several different mycobacterial antigens that have been purified and identified mainly by affinity chromatography on monoclonal antibody columns or by recombinant gene expression, some of which have been indicated to be valuable in myco-bacterial serology (11, 25 and 26). In 2009, Meikle and colleagues reported detection *M. bovis* antigens through a screening of native protein fractions from a culture of *M. bovis* AN5. They separated antigenic fractions into individual proteins by two-dimensional (2D) gel electrophoresis (2D-E). They also reported thirty protein fractions in various concentrations from 17 to 80 μg/ml and with molecular weights ranging from 7 to 208 kDa (3). By contrast in present investigation, the antigenic proteins were isolated, purified by Sephadex G50 chromatography and immunologically characterized. Furthermore, we analyzed three main fractions contained protein concentrations ranging from 14 to 60 μg/ μl with molecular weights of 10 to 180 kDa. While, significantly the third purified fraction (F3) was in a broader range of 10 to 40 kDa showing a single pronounced band with two minor bands with slight size differences on SDS-PAGE. In 1996, Bassey et al. reported predominance of low molecular weight fractions with immune reactivity. They pronounced the presence of strong antigens, such as the 6 kDa early secretory antigenic target ESAT-6 and 10 kDa culture filtrate antigen CFP-10, in low-molecular-weight fractions, because their predominance was also seen when the fraction was prepared with *M. bovis* BCG in the absence of these antigens (26). In 1987, Lamb and Young reported similar predominance. They reported T-cell recognition of antigens fractionated by SDS-PAGE and added to proliferation assays after blotting onto nitrocellulose membranes (27). These researchers discriminated between the responses to culture filtrate and cell extracts.

As observed by a number of authors, proteins from the culture supernatant are known to be more reactive than those from the cell extract, and many strongly reacting antigens (Ag85 complex, ESAT-6, CFP-10, MPB70, MPB83, P38, etc.) of *M. tuberculosis* complex proteins were reported to be present in the supernatant fluid (24–29). Similarly we were also able to isolate proteins of various molecular sizes below 40 kDa, they could be crucially P38, ESAT-6 or CFP-10.

During present study the target antigen was identified by dot-blot. In contrast to the conventional bacteriological methods, the dot-blot is less time consuming and is more precise. In 1999, Sumi and coworkers investigated rapid diagnosis of tuberculous meningitis (TBM) by dot-blot to discover mycobacterial antigen in cerebrospinal fluid specimens (CSF) (21). In accomplished dot-blot, positive reactions with *M. bovis* infected cattle serum (polyclonal antibody) were visible in this study and it can prompt antigen detection. The results were reproducible with estimated specificity of the assay.

Additionally, in the present study we used western blotting to analyze the specificity of the purified protein fractions against the sera of infected cattle. In 2005, Raju et al. reported that the antibody response to the 38 kDa, 16 kDa and Lipoarabinomannan (LAM) antigens of *M. tuberculosis* evaluated using western blot. According to their results, they detected an antibody response to *M. tuberculosis* proteins in the range of 150 kDa to 16 kDa, while 84–89 kDa protein were the major protein bands observed in 73.3% of cases (22). Munk and coworkers (1988) in their investigation used polyclonal antibodies and observed high levels of non-specific reactions bands on membrane (30). In contrast to these reports, it is interesting that in our study we were able to achieve significant results with the serum of *M. bovis* infected cattle. The serum sample was utilized as a source of polyclonal antibody and no non-specific reactions were observed. Polyclonal antibodies were used in this study mainly because it is considered that monoclonal antibody might be too specific to react with the obtained antigenic fractions. Negative serum samples were also used during western blotting analysis to evaluate the specificity and sensitivity of the purified protein fraction. No obvious bands on negative controlled membrane indicated the specificity of the purified antigens.

## CONClUSION

This research aimed to identify and purify antigenic proteins from culture filtrates of *M. bovis* AN5 for use in immunological assays. These antigens used in serological and cellular response studies could improve the diagnostic capability of the immunological methods such as ELISA, the IFN-γ assay and/or further vaccine development for either bovine or human disease and has the potential to enhance the specificity of diagnostic tests. It is advisable to test the obtained and purified antigens in animals.
